# Cross-comparison of MRCGP & MRCP(UK) in a database linkage study of 2,284 candidates taking both examinations: assessment of validity and differential performance by ethnicity

**DOI:** 10.1186/s12909-014-0281-2

**Published:** 2015-01-16

**Authors:** Richard Wakeford, MeiLing Denney, Katarzyna Ludka-Stempien, Jane Dacre, I C McManus

**Affiliations:** Hughes Hall, University of Cambridge, Cambridge, CB1 2EW UK; Royal College of General Practitioners, 30 Euston Square, London, NW1 2ED UK; UCL Medical School, University College London, Gower Street, London, WC1E 6BT UK; Research Department of Clinical, Educational and Health Psychology, University College London, Gower Street, London, WC1E 6BT UK

**Keywords:** MRCGP, MRCP(UK), Applied knowledge test, Clinical skills assessment, PACES, Correlation, Ethnicity, Black and minority ethnic

## Abstract

**Background:**

MRCGP and MRCP(UK) are the main entry qualifications for UK doctors entering general [family] practice or hospital [internal] medicine. The performance of MRCP(UK) candidates who subsequently take MRCGP allows validation of each assessment.

In the UK, underperformance of ethnic minority doctors taking MRCGP has had a high political profile, with a Judicial Review in the High Court in April 2014 for alleged racial discrimination. Although the legal challenge was dismissed, substantial performance differences between white and BME (Black and Minority Ethnic) doctors undoubtedly exist. Understanding ethnic differences can be helped by comparing the performance of doctors who take both MRCGP and MRCP(UK).

**Methods:**

We identified 2,284 candidates who had taken one or more parts of both assessments, MRCP(UK) typically being taken 3.7 years before MRCGP. We analyzed performance on knowledge-based MCQs (MRCP(UK) Parts 1 and 2 and MRCGP Applied Knowledge Test (AKT)) and clinical examinations (MRCGP Clinical Skills Assessment (CSA) and MRCP(UK) Practical Assessment of Clinical Skills (PACES)).

**Results:**

Correlations between MRCGP and MRCP(UK) were high, disattenuated correlations for MRCGP AKT with MRCP(UK) Parts 1 and 2 being 0.748 and 0.698, and for CSA and PACES being 0.636.

BME candidates performed less well on all five assessments (P < .001). Correlations disaggregated by ethnicity were complex, MRCGP AKT showing similar correlations with Part1/Part2/PACES in White and BME candidates, but CSA showing stronger correlations with Part1/Part2/PACES in BME candidates than in White candidates.

CSA changed its scoring method during the study; multiple regression showed the newer CSA was better predicted by PACES than the previous CSA.

**Conclusions:**

High correlations between MRCGP and MRCP(UK) support the validity of each, suggesting they assess knowledge cognate to both assessments.

Detailed analyses by candidate ethnicity show that although White candidates out-perform BME candidates, the differences are largely mirrored across the two examinations. Whilst the reason for the differential performance is unclear, the similarity of the effects in independent knowledge and clinical examinations suggests the differences are unlikely to result from specific features of either assessment and most likely represent true differences in ability.

## Background

### The validity and fairness of assessments

Assessments of all sorts, particularly high-stakes assessments, need to be valid. The meaning of ‘validity’ has evolved over the decades, and a recent review emphasizes that “test scores are of interest because they are used to support claims that go beyond (often far beyond) the observed performances” [1] (p.1). That is certainly the case for post-graduate medical examinations, where passing an examination provides entry into a specialist career, and failure means the abandonment of that career route.

Validity is a difficult concept, with many definitions and sub-categories, and changing ideas about its interpretation [1-4]. The AERA/APA/NCME *Standards for Educational and Psychological Testing* of 1999 [4] stress the fundamental nature of validity for any test, and say that, “a sound validity argument integrates various strands of evidence into a coherent account of the degree to which existing evidence and theory support the intended interpretation of test scores for specific uses” (p.17). In this paper one of our aims is to concentrate on just one of those strands, one which has hardly been looked at for UK postgraduate assessments, which is the extent to which performance on one assessment correlates with subsequent performance on another. For practical reasons it is rare for specialist examinations in different specialties to be taken by the same candidates. Nevertheless we have found a substantial cohort of individuals who have taken both MRCGP and MRCP(UK), two separate postgraduate examinations, which have different syllabuses, different methods of measurement, and are run by entirely separate organizations. That group allows comparison of performance on the two separate examinations and as such is a form of concurrent validity, albeit that one assessment is taken somewhat later than the other.

This is not the place to articulate the wider argument for the validity of postgraduate medical examinations specifically, or of school-level or undergraduate examinations more generally, which is complex, but we note a) that there is a continual chain of correlations across school-level, undergraduate and postgraduate assessments, which we have called the ‘academic backbone’ [5]; and b) that clinical outcomes are correlated with performance on postgraduate examinations (as seen in a study in Québec, where higher scores on licensing examinations correlated with better clinical family practice in terms of screening and prescribing behaviours [6], and in a US study in which higher scores at USMLE Step 2 CS were associated with lower mortality from acute myocardial infarction and congestive cardiac failure [7]. Together those, and other such studies and arguments, suggest that postgraduate examinations in general are probably valid predictors of behaviour in actual clinical practice.

Issues of validity are closely tied up with issues of fairness. If a test score can be interpreted as valid, then differences in performance between different groups of doctors can be considered to represent true differences in ability, and hence the examination can be seen as fair despite group differences.

Interest in the fairness of assessments of doctors has been greatly heightened in the UK during 2013–2014 because of a High Court challenge (‘Judicial Review’) against the Royal College of General Practitioners in connection with their clinical OSCE, the Clinical Skills Assessment (CSA) [8]. The CSA is taken at the end of specialist training for General Practice (GP) and is seen as a critical ‘exit examination’. An organization representing ethnic minority doctors (BAPIO: the British Association of Physicians of Indian Origin) had asked the Court to consider its claim that the College was unlawfully discriminating against Black and Minority Ethnic (BME) doctors in the CSA, both directly and indirectly.

The legal challenge was in the event rejected by the Court in April 2014 with the Judge concluding that the CSA was a proportionate means of achieving a legitimate aim and that it was neither directly nor indirectly discriminatory; he stated that there was “no basis for contending that the small number who fail [the CSA] ultimately do so for any reason apart from their own shortcomings as prospective general practitioners” [8]. Overall costs of the case were substantial, in the order of £½ M.

Many UK medical assessments, both at undergraduate and postgraduate level, show differences in performance according to ethnicity [9], including both MRCP(UK) [10], and MRCGP (where the issues has been flagged in annual reports since 2008, and in the current report [11]). During 2013, this matter had become more controversial in the case of the MRCGP, in part due to increasing public agitation by BAPIO, and as a result the GMC instigated a review, a report on which was published by that body in September 2013 [12], along with a separate paper in the BMJ with somewhat different conclusions [13].

In 2013, the GMC also instigated two other studies which have become relevant to considerations of performance by ethnicity; these had the main aim of reviewing the ‘equivalence’ of international medical graduates (IMGs) to UK graduates in their performance on two postgraduate assessments (MRCGP, MRCP(UK)) and in the Annual Review of Competency Panels (ARCP), conducted by the UK postgraduate training deaneries. Reports on these studies were published in April 2014, shortly after the Judicial Review ended [14,15]. They showed that the performance of IMGs in the UK was well below that of UK graduates on all available comparative measures, and that if equivalent performance on these measures was desired, then the level of the entry tests for IMGs wishing to practice in the UK, those of the GMC’s Professional Linguistics and Assessment Board (PLAB), would need to be raised substantially. Candidates’ ethnicity is of course strongly confounded with primary medical qualification (PMQ), a majority of IMGs coming from ethnic minorities. It is also the case that IMGs underperform in other countries than the UK, including Australia [16,17].

Whether or not the MRCGP examination components actually correlate with the MRCP(UK) assessments is clearly of particular interest to the RCGP. With the completion of the Court case and the publication of the papers on PLAB, it is important and now realistic to explore the issue of differential performance publicly and dispassionately. Having information about candidates’ performance on two separate assessments allows one way of doing this.

### Aims of the present study

The first aim of this paper is to evaluate the general extent to which the performance of candidates on one examination predicts their performance on the other, which may be seen as indicating the extent of an aspect of their validity. High-stakes, postgraduate medical assessments should be valid. However attempts to provide formal evidence of validity are, in practice, rare, for a host of reasons. In medicine, the scarcity of such data reflects the fact that there is no national UK qualifying examination taken by all graduates, and that relatively few doctors who are training in one specialty will subsequently take exams in another specialty.

The second, more specific, aim of this paper is to examine the performance of those candidates of different ethnicities who, unusually amongst UK doctors, sat the entirely separate assessments of two major examining bodies. Did they fare similarly under each?

### The assessments

Both MRCGP and MRCP(UK) have knowledge assessments (MRCGP AKT (Applied Knowledge Test), and MRCP(UK) Part 1 and Part 2, all being conventional multiple choice assessments), and each also has a clinical assessment (PACES (Practical Assessment of Clinical Examination Skills) for MRCP(UK) and CSA (Clinical Skills Assessment) for MRCGP, both being variants of an OSCE assessment. Candidates who take both assessments have typically undertaken the MRCP(UK) during the years immediately after qualification, and , and the MRCGP AKT in the second year of their three-year GP training programme and the CSA in their final year.

Although general practice medicine and hospital medicine are different specialties, inevitably both of them share various components, reflecting the nature of disease, its presentation, its ætiology, its diagnosis, and its treatment. There is therefore a reasonable expectation that doctors who perform better at, say, MRCGP will also perform better at MRCP(UK), and vice-versa. The examinations also each have knowledge and clinical assessments, and it seems reasonable to predict that there will be some congruence on those assessments. Our analysis therefore looked not only at overall performance on the examinations, but also at performance on the different components (and in the case of the MRCGP, at the three separate marks which are available within the AKT).

MRCGP and MRCP(UK) are examinations with moderately high failure rates, and hence candidates can, and often do, re-sit the assessments on a number of times. Previous detailed analyses of MRCP(UK) have shown that the mark at the first attempt is the best correlate of performance at other components of the exam, and it also predicts subsequent performance at the exam [18], and hence all analyses here are restricted to marks at first attempts.

## Methods

Separate databases were available for the MRCGP and MRCP(UK) examinations.

### MRCGP

Data for MRCGP AKT and CSA were available from October 2007 until May 2011; since the two components are typically taken a year apart, there were some candidates for whom data was available only for one component.

The AKT is sub-divided into three sections, for each of which a separate score is available: at that time, 80% of question items were on clinical medicine, 10% on critical appraisal and evidence based clinical practice, and 10% on health informatics and administrative issues. Standard-setting was by means of an Angoff process, with statistical equating across diets.

The CSA exam changed somewhat in autumn 2010, with the approval of the GMC. Previously, although marked on each of the (then, twelve) cases on three criteria of data-gathering, clinical management and interpersonal skills, candidates were separately adjudged ‘pass’ or ‘fail’ on each station, and to pass the examination they needed to achieve a specific number of ‘passes’ (normally, eight out of twelve). From the 2010, the number of cases was increased to thirteen and the standard-setting process was changed to the borderline group method, calculated on a daily basis [19]. The ‘new’ total mark comprises a summation of the three domain scores (each 0—3) on the thirteen cases making the total mark out of 117.

Because of a varying pass mark on a daily (CSA) or diet (AKT) basis, all candidates’ scores are scaled to a standard pass-mark of zero for reporting purposes.

### MRCP(UK)

The data for MRCP(UK) consisted of a ‘history file’ extracted on 10^th^ November 2011, containing information on candidates taking MRCP(UK) Part 1, Part 2 and PACES from 2003, 2002 and 2001 onwards. Part 1 and Part 2 were originally standard-set using a modified Angoff technique combined with a Hofstee method, but from the diets of 2008/3 and 2010/1 respectively until the present have been set using statistical equating based on Item-Response Theory (IRT), with a one-parameter IRT (Rasch) model [20]. The presentation of marks changed at the same time, and all marks are presented in the current marking scheme which was equated to a mean of 500 and a standard deviation of 100 for the reference cohort used for equating, with earlier marks being put onto the new scheme. The marking scheme for PACES changed in 2009 [21], although almost all of the present candidates had in fact taken it before then. Although the current marking scheme for PACES has seven skills, with a pass being required in each, a total mark is also available for statistical purposes and it has been used here for the 63 candidates (6.7%; n = 944) taking the new PACES, with earlier diets being equated to the present marking scheme. Marks for Part 1, Part 2 and PACES are expressed relative to the pass mark, positive or zero marks meaning a candidate passes and negative marks that they fail.

### Linkage

All candidates taking MRCGP are on the UK medical register (LRMP; List of Registered Medical Practitioners), and hence have a GMC number. MRCP(UK) candidates can take the examination outwith the UK, and many will not have a GMC number, but neither will they be taking MRCGP. Linkage of the MRCGP and MRCP(UK) databases was thus by means of the GMC number.

### Statistical analysis

Statistical analysis used IBM SPSS 21.0.

### Ethical permission

University College London has confirmed that analysis of educational data such as those in the present analysis is exempt from requiring ethical permission (see http://ethics.grad.ucl.ac.uk/exemptions.php).

## Results

The MRCGP database contained information on 8,919 candidates who had taken either AKT and/or CSA. The MRCP(UK) database had information on 53,603 candidates who had taken one or more of Part 1, Part 2 and PACES, with GMC numbers available for 31,909 candidates. The merged file contained information on 2,284 candidates who had taken one or more parts of both MRCGP and MRCP(UK). MRCGP AKT was taken an average of 3.7 years (SD 1.33 years) after MRCP(UK) Part 1, with only 1.0% of candidates taking AKT before Part 1. Analyses not reported here suggest that the order of taking MRCGP and MRCP(UK) did not correlate with AKT, but those who took MRCP(UK) first were somewhat more likely to pass CSA [22].

### Descriptive statistics

Doctors who take both MRCGP and MRCP(UK) may well not be representative of those taking either exam in general, with aspirationally ‘high-flying academic GPs’ perhaps wishing to pass MRCP(UK) before entering general practice training, and this latter group they may have taken all three parts of MRCP(UK). Alternatively, the much larger group of doctors who took MRCP(UK) Part 1, but then did not go on to take the other parts of MRCP(UK) may have been discouraged from a career in hospital medicine by their performance on Part 1, and may have therefore turned to general practice.

Table 1 summarises the overall performance of different groups of candidates to assess the extent to which candidates differ in their overall ability, and in particular it compares the group who have taken both MRCP(UK) and MRCGP with candidates in general taking either assessment.

**Table 1:** **Descriptive statistics for the MRCP(UK) and MRCGP components**

**(a)**	**(b)**	**(c)**	**(d)**	**(e)**	**(f)**
**Assessment component**	**All candidates taking MRCGP**	**All candidates taking MRCP(UK) [See notes]**	**Candidates taking at least one part of both MRCGP and MRCP(UK)**	**Candidates who have taken all three parts of MRCP(UK)**	**Candidates taking either part of MRCGP and all three parts of MRCP(UK)**
MRCGP AKT	**16.03** (15.69) N = 8919	-	**18.11** (14.79) N = 2284	**-**	**25.67** (11.9) N = 741
MRCGP CSA	**9.39** (12.89) N = 2634	-	**5.93** (13.91) N = 564	**-**	**8.81** (13.43) N = 185
MRCP(UK) Part 1	-	**−28.93** (130.6) N = 23344	**−62.48** (119.47) N = 1988	**14.98** (110.7) N = 11039	**6.14** (101.6) N = 741
MRCP(UK) Part 2	-	**47.45** (87.38) N = 17008	**26.00** (75.62) N = 1131	**55.85** (83.34) N = 11039	**42.6** (71.5) N = 741
MRCP(UK) PACES	-	**-.18** (21.97) N = 17806	**−2.54** (22.2) N = 943	**1.33** (21.72) N = 11039	**−1.04** (21.9) N = 741

*Notes:* For each assessment (column a), statistics are shown for all of those taking MRCGP (column b) and MRCP(UK) (column c), for those taking at least one part of each assessment (column d), for those taking all three parts of MRCP(UK) (column e) and for those taking all three parts of MRCP(UK) and at least one part of MRCGP (column f). For MRCP(UK), ‘All candidates’ refers to all candidates in the database with a GMC number and who have therefore probably worked in the UK.

- Column (b) shows the performance of *all* candidates in the MRCGP database;
- Column (c) shows the performance of *all* candidates in the MRCP(UK) database:
- Column (d) shows the mean scores of those doctors who had taken *at least one* part of *both* MRCGP and MRCP(UK): it can be seen that performance was above average for AKT, but below average for CSA, and the three parts of MRCP(UK)
- Column (e) shows the performance of all doctors in the MRCP(UK) database who had taken all three parts of the examination (and hence had passed Parts 1 and 2)
- Column (f) shows the performance of all doctors who had taken MRCGP *and all three parts of MRCP(UK)*: while they performed better at AKT and a little worse at CSA, they performed better overall than typical candidates at MRCP(UK), but somewhat less well than the candidates in Column (e)

### Correlations overall

Table 2 shows the Pearson correlations (r) between the marks on MRCP(UK) Parts 1, 2 and PACES, and MRCGP AKT (including the sub-marks for clinical medicine, evidence interpretation and organisational questions), and CSA (including separate analyses for the old and the new format).

**Table 2:** **Correlations between performance on MRCP(UK) and MRCGP components**

	**MRCP(UK) Part 1**	**MRCP(UK) Part 2**	**MRCP(UK) PACES**
**MRCGP AKT**	**r = .673**	**r = .600**	**r = .471**
**Total mark**	**p < .001**	**p < .001**	**p < .001**
	**N = 1,988**	**N = 1,131**	**N = 943**
	**r**_**d**_ **= .748**	**r**_**d**_ **= .698**	**r**_**d**_ **= .555**
MRCGP AKT	r = .662	r = .570	r = .438
Clinical Medicine	p < .001	p < .001	p < .001
	N = 1,988	N = 1,131	N = 943
MRCGP AKT	r = .457	r = .425	r = .369
Evidence interpretation	p < .001	p < .001	p < .001
	N = 1,988	N = 1,131	N = 943
MRCGP AKT	r = .314	r = .319	r = .223
Organisational questions	p < .001	p < .001	p < .001
	N = 1,988	N = 1,131	N = 943
**MRCGP CSA**	**r = .348**	**r = .386**	**r = .496**
**Total mark**	**p < .001**	**p < .001**	**p < .001**
	**N = 1,988**	**N = 1,131**	**N = 943**
	**r**_**d**_ **= .421**	**r**_**d**_ **= .489**	**r**_**d**_ **= .636**
MRCGP CSA	r = .315	r = .368	r = .473
Old Format (to May 2010)	p < .001	p < .001	p < .001
	N = 1,479	N = 849	N = 717
MRCGP CSA	r = .430	r = .440	r = .582
New Format (Sep 2010 onwards)	p < .001	p < .001	p < .001
	N = 509	N = 282	N = 226

Notes: Correlations are given as between the three parts of MRCP(UK) (columns) with performance at the MRCGP AKT (overall, in bold; and with its three sub-scores), and with the MRCGP CSA, overall, in bold; and separately for the old and new format CSA. Conventional, empirical correlations (r) are shown at the top of each cell, whereas correlations disattenuated for unreliability (r_d_) are shown at the bottom of the main cells.

Considering just the main correlations, shown in bold, they are highly significant (all p < .001) between the two parts of MRCGP and the three parts of MRCP(UK). Looking in more detail, it is clear that AKT correlates most highly with MRCP(UK) Part 1 (.673), a little less with Part 2 (.600), and least of all with PACES (.471), although the latter correlation is still substantial and highly significant. CSA shows the inverse pattern of correlations, highest with MRCP(UK) PACES (.496), somewhat less with Part 2 (.368) and least of all with Part 1 (.348), although again the latter is highly significant.

The results can be summarised succinctly as knowledge tests correlating better with knowledge tests and clinical tests with clinical tests. That pattern is supported by the sub-tests of AKT, where clinical medicine correlates most highly with MRCP(UK) Part 1 (and Part 1 is almost entirely about clinical medicine and the applied biomedical science underlying it), somewhat less with evidence interpretation, and least of all with organisational aspects (which relate particularly to NHS general practice).

The old and the new formats of CSA show broadly similar patterns of correlation with MRCP(UK), although it is clear that the new format CSA has *higher* correlations overall – perhaps unsurprisingly in view of the ‘finer granularity’ of the mark (0—117 as opposed to 0–12). Without giving significance tests for all cases, the one with the smallest Ns (and hence the least powerful) is the comparison of the correlations of PACES with old and new CSA, where the difference using Fisher’s Z test is significant (z = 1.976, two-tailed p = .0482). Other comparisons are more significant.

### Disattenuation of correlations

The correlations shown at the top of each cell in Table 2 are conventional empirical correlations (‘r’). However, all measurements, be they of examinations or other behavioural measures, have error and hence are unreliable to a greater or lesser extent. In order to interpret correlations, particularly when examinations differ in their reliability, it is helpful to ‘disattenuate’ them for differences in reliability. Disattenuated correlations give a more accurate estimate of the shared variance between two tests (*r*_*d*_^*2*^).

Reliability coefficients for MRCGP and MRCP(UK) have been estimated, and are about .89 for MRCGP AKT and .75 for MRCGP CSA, and for MRCP(UK) are about .91 for Part 1 [23,24], .83 for Part 2 [24], and .81 for PACES [25]. In Table 2, the bottom line of the main entries shows the value of *r*_*d*_, the disattenuated correlation which takes account of measurement error using the standard formula *r*_*d*_ *= r*_*12*_*/sqrt(r*_*11*_*x r*_*22*_*),* where *r*_*11*_ and *r*_*22*_ are the reliabilities of the two variables for which r_12_ is the conventional correlation. The pattern of correlations remains similar, with the knowledge tests predicting other knowledge tests and the clinical measures predicting the clinical measures.

### Ethnicity and performance

Ethnicity data were available for both the MRCGP and MRCP(UK) databases, although these did not always concur, being self-reported and self-entered data. Ethnicity was therefore classified as white if the candidate had declared themselves as white in both of the databases, and otherwise was classified as BME. Of the 2,284 candidates who had taken both examinations, 854 (37.4%) were white and 1,430 (62.6%) were BME. 1,401 (61.3%) of the 2,284 candidates were graduates of UK medical schools, of whom 600 (42.8%) were BME, whereas of the 883 non-UK graduates, 830 (94.0%) were BME. The analyses described below for all candidates have been repeated for UK graduates alone and almost identical results have been found, and therefore results regarding ethnicity will be reported here for the full candidate group.

Studies elsewhere [10,26,27] have shown that BME candidates underperform at MRCGP and MRCP(UK), and those effects are also found in the present data, BME candidates in the present sample performing less well at MRCP(UK) Part 1, Part 2 and PACES, and at AKT, its three subtests, and CSA (detailed results will not be presented but all are p < .001). Understanding the mechanism is not straightforward, but having both MRCP(UK) and MRCGP data allows an additional handle on the problem, and we know of no other studies that examine performance in terms of this variable which look at the relationship between two examinations.

Table 3 summarises a series of multiple regression analyses, assessing the effect of ethnicity (BME) after taking earlier performance into account. Effects are reported in terms of ‘b’ and ‘beta’. ‘b’ coefficients are simple regression coefficients, a value of ‘b’ indicating that for an increase of one unit on the scale of the (independent) predictor variable there is a change of b units on the outcome (dependent) variable. ‘b’ coefficients are useful for comparing across groups, particularly when the standard deviation of groups may vary. The ‘beta’ coefficients are standardised ‘b’ coefficients, and show for a one standard deviation increase in the predictor variable the number of standard deviations by which the outcome variable will change. ‘b’ coefficients are on the units of the outcome variable, and therefore are not easily compared across different assessments, whereas beta coefficients are dimension-less (like correlation coefficients) and hence can be more readily compared across different assessments.

**Table 3:** **Assessment of ethnic differences in performance, expressed as the relative performance of BME candidates**

**Dependent variable**	**Variables taken into account**	**BME effect**
MRCP(UK) Part 1	-	beta = −.265, p < .001, b = −64.70
		N = 1988
MRCP(UK) Part2	MRCP(UK) Part 1	beta = −.163, p < .001, b = −24.95
		N = 942
MRCP(UK) PACES	MRCP(UK) Part 1 & Part2	beta = −.240, p < .001, b = −10.55
		N = 741
MRCGP AKT	MRCP(UK) Part 1, Part 2 & PACES	beta = −.127, p < .001, b = −3.03
		N = 741
MRCGP CSA	MRCGP AKT; MRCP(UK) Part 1, Part 2 & PACES	beta = −.208, p < .001, b = −5.11
		N = 741
MRCGP AKT - CM	Part1, Part2, PACES	beta = −.105, p = .001, b = −1.23
(Clinical Medicine)		N = 741
MRCGP AKT – EI	Part1, Part2, PACES	beta = −.079, p = .024, b = −2.21
(Evidence Interpretation)		N = 741
MRCGP AKT – O	Part1, Part2, PACES	beta = −.123, p = .001, b = −3.02
(Organisational)		N = 741
MRCGP AKT - CM	MRCP(UK) Part 1, Part 2 & PACES; MRCGP AKT-EI & AKT-O	beta = −.075, p = .01 , b = −0.88
(Clinical Medicine)		N = 741
MRCGP AKT – EI	MRCP(UK) Part 1, Part 2 & PACES; MRCGP AKT-CM & AKT-O	beta = −.029, NS (p = .393) , b = −0.78
(Evidence Interpretation)		N = 741
MRCGP AKT – O	MRCP(UK) Part 1, Part 2 & PACES; MRCGP AKT-CM & AKT-EI	beta = −.087, p = .015, b = −2.13
(Organisational)		N = 741

*Notes*: For the various assessments shown in the first column, the effects are shown in the third column, after taking into account performance on previous assessments, shown in the second column. A negative beta or b coefficient indicates that BME candidates perform less well than White candidates.

The multiple regressions show that MRCP(UK) Part 1, the examination almost always taken first, shows a strong effect of ethnicity. Part 2 also shows an effect of ethnicity, after taking Part 1 into account; and similarly PACES shows an ethnicity effect after taking Parts 1 and 2 into account. MRCGP shows a similar pattern, AKT showing an ethnicity effect after taking all three parts of MRCP(UK) into account, and CSA showing a similar ethnicity effect after taking MRCP(UK) and AKT into account. Similarly the sub-scores on AKT also show an ethnicity effect, and with the exception of AKT evidence Interpretation, the AKT sub-scores shown ethnicity effects even after taking other sub-scores into account.

### Ethnicity and the CSA

The relationship between MRCP(UK) performance and MRCGP performance was assessed separately for the total AKT and CSA marks, and the Part 1, Part 2 and PACES marks (see Table 4), correlations being calculated separately for white (‘W’) and BME candidates who had taken the old or the new CSA assessment.

**Table 4:** **Correlation of performance on the three parts of MRCP (UK) and on the total scores at MRCGP AKT and CSA, by ethnicity**

**Correlation with MRCGP AKT or CSA**	**Ethnicity**	**CSA Format**	**MRCP(UK) Part 1**	**MRCP(UK) Part 2**	**MRCP(UK) PACES**
**AKT Total Mark**	BME	Old	.654*** N = 872	.560*** N = 463	.423***N = 386
		New	.607*** N = 342	.558*** N = 203	.383*** N = 165
		Old vs New	P = .223	P = .973	P = .610
		**All CSA**	.636*** N = 1214	.558*** N = 666	.411*** N = 551
	White	Old	.643*** N = 607	.543*** N = 386	.352*** N = 331
		New	.696*** N = 167	.575** N = 79	.231 NS N = 61
		Old vs New	P = .274	P = .711	P = .352
		**All CSA**	.655*** N = 774	.550*** N = 465	.331*** N = 392
	**BME vs W**	**Old and New**	**P = .502**	**P = .849**	**P = .161**
	All	Old	.682*** N = 1479	.600*** N = 849	.478*** N = 717
	All	New	.655*** N = 509	.592*** N = 282	.427*** N = 226
	All	Old vs New	P = .343	P = .857	P = .403
**CSA Total Mark**	BME	Old	.254*** N = 872	.306*** N = 463	.415*** N = 386
		New	.405*** N = 342	.422*** N = 203	.537*** N = 165
		Old vs New	*P = .008*	P = .114	P = .091
		**All CSA**	.299*** N = 1214	.341*** N = 666	.453*** N = 551
	White	Old	.199*** N = 607	.245*** N = 386	.279*** N = 331
		New	.305*** N = 167	.270* N = 79	.457*** N = 61
		Old vs New	P = .198	P = .831	P = .146
		**All CSA**	.209*** N = 774	.234*** N = 465	.288*** N = 392
	**BME vs W**	**Old and New**	***P = .037***	***P = .054***	***P = .004***
	All	Old	.320*** N = 1479	.369 *** N = 849	.467 *** N = 717
	All	New	.430*** N = 509	.440 *** N = 282	.582 *** N = 226
	All	Old vs New	*P = .013*	*P = .218*	*P = .038*

*Notes*: Correlations are shown separately for White and BME candidates, taking CSA in the old and in the new format. Significances of correlations are shown as ***p < .001; **p < .01; *p < .05; NS Not significant. Significant P values in comparisons are shown in italics.

Old and new CSA were compared for the twelve combinations of White/BME × Old/NewCSA × AKT/CSA × Part1/Part2/PACES, of which only one was significant with p = .008. Overall, the old CSA and the new CSA seem to be performing in a broadly equivalent way. The six comparisons of Old/New CSA × AKT/CSA × Part1/Part2/PACES, merging White and BME were significant in only two cases (see bottom of Table 4: p = .013 and p = .038).

Correlations between AKT/CSA and Part1/Part2/PACES were compared for White and BME candidates, merging across the old and the new CSA. AKT showed similar correlations with Part1/Part2/PACES in White and BME candidates (p = .502, .849 and .161). However, CSA showed stronger correlations with Part1/Part2/PACES in BME candidates than in White candidates (p = .037, .054 and .005).

Altogether, 21 pairs of correlations have been compared, of which six were significant (28%) which is somewhat more than the 5% expected by chance alone. The pattern is not completely clear, but is certainly not compatible with the new CSA being less valid than the old CSA, nor with the CSA and AKT correlating less in BME than white candidates, and in both cases the pattern may be significant in the opposite direction.

**Figure 1:**
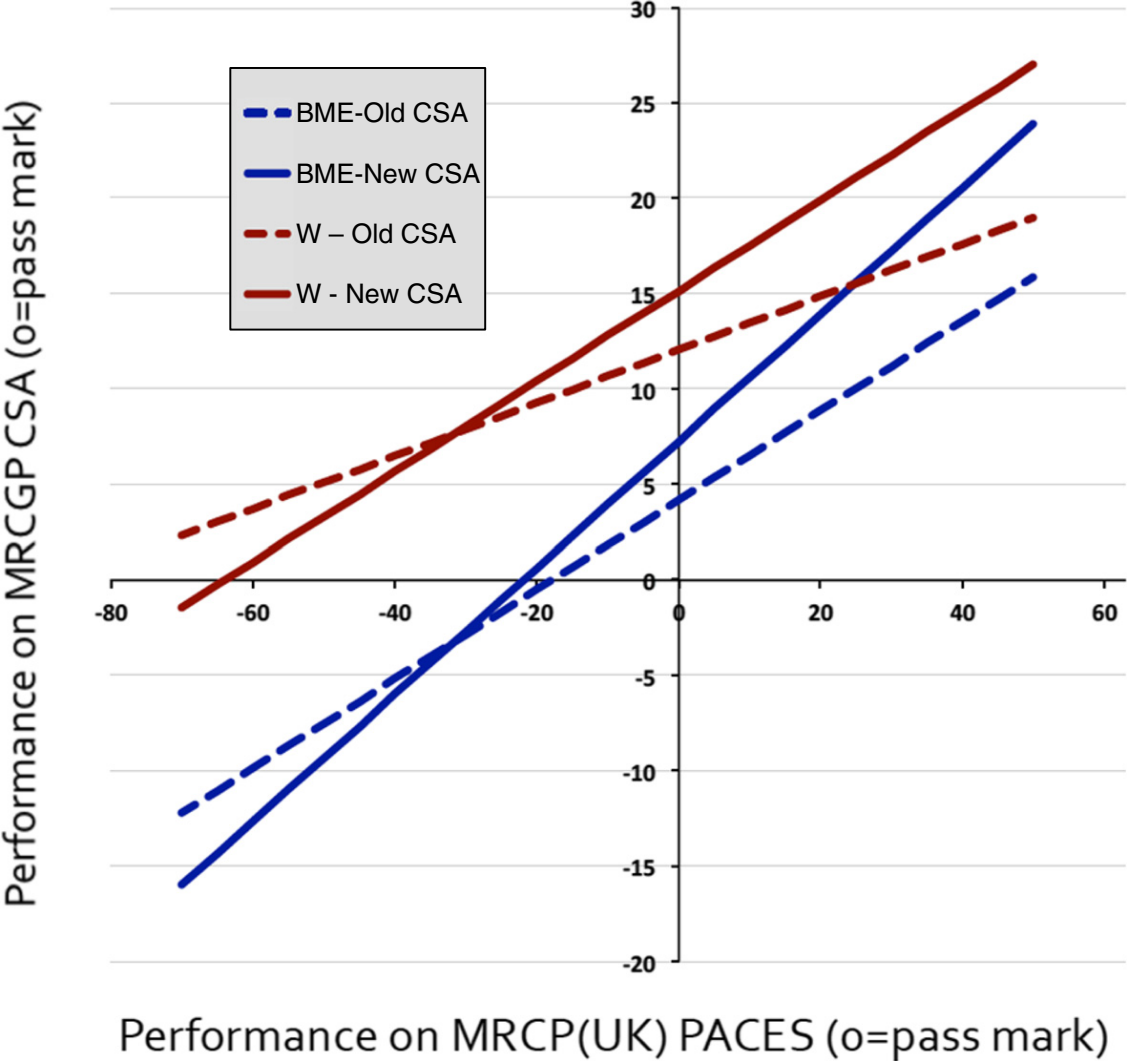
**Performance of candidates on MRCGP CSA in relation to previous performance on MRCP(UK) PACES, by ethnicity.** Data are summarized for White (W) and BME candidates taking the Old or the New version of CSA. Lines shown are fitted lines from multiple regression.

Performance on the CSA was explored further using multiple regression (see Figure 1), with CSA performance as the dependent variable, and a series of predictors, including PACES performance, BME and CSA type (old vs new) and their interactions. Interaction terms involving PACES influence the slope of the lines, whereas terms not involving PACES influence the intercept (i.e. the height of the lines), and because of the way PACES is coded, the intercepts can be interpreted as differences in performance on CSA of those who are exactly on the pass mark for PACES. Forward-entry regression was used, with lower order terms being entered before higher-order terms. PACES was the most significant predictor of CSA performance (n = 741, beta = .465).

Intercepts for BME and CSA type were both significant (p < .001), but there was no significant effect on intercept of BME × CSA type. The slopes differed significantly for PACES × BME and PACES × CSA type, but there was not a significant PACES × BME × CSA type interaction. Slopes were lower in white candidates (b = −.095, p = .009) and were higher for the new CSA assessment (b = .098, p = .007).

Figure 1 shows the fitted effects for the relationship between CSA and PACES in the four sub-groups. The intercepts are for the point where the lines cross the vertical line indicating a PACES score of zero (i.e. the pass mark). The slope is highest in BME candidates taking the new CSA, and lowest in the white candidates taking the old CSA.

Taken overall, Figure 1 shows that PACES performance is a good predictor of CSA performance, and that the new CSA is being predicted better than was the old CSA, implying an increase in the validity of the new CSA over the old. BME candidates perform less well on the CSA, even after taking PACES performance into account, but the size of that difference is the same for the new CSA and the old CSA, suggesting that the new CSA is not treating BME candidates differently to White candidates.

## Discussion

A comparison of the performance of candidates who have taken both MRCGP and MRCP(UK) assessments helps in understanding a number of issues concerning the validity of both of the examinations, as well as the impact of other factors such as ethnicity and the change in the CSA assessment. Before considering the issue of performance by ethnicity, it is helpful to reflect on the general relationship between the two sets of assessments and upon the demographics of candidate taking each.

### Correlations between MRCGP and MRCP(UK) assessments

There is little doubt that performance at MRCGP and MRCP(UK) are substantially correlated, with disattenuated correlations of .748 and .698 between the knowledge exams, and .636 between the clinical assessments. Candidates who do better at one exam therefore do better at the other, even though MRCGP is typically taken more than three years later than MRCP(UK). The two examinations do not include the same questions or the same technical material, although there is inevitably overlap in the broad domains of medical knowledge being assessed - the GP curriculum is broader, including coverage of clinical areas of obstetrics and gynaecology, psychiatry, otorhinolaryngology etc., whereas that of the physicians will cover the different medical topic areas in greater depth.

Because MRCGP is usually taken after MRCP(UK), the correlation may reflect material that has been learned for MRCP(UK) subsequently being useful for the MRCGP examination. That would be supported, but only partly supported, by Part 1 correlating most highly with the clinical medicine component of AKT and least with the organisational questions (material which does not appear in MRCP(UK)). However the latter correlation is still .314 and highly significant, and therefore the association may reflect some other shared mechanism, perhaps of motivation, approaches to study, or prior scientific and clinical knowledge, all of which have been argued to contribute to the ‘academic backbone’ [5] which underpins school-level, undergraduate and postgraduate performance, performance at each stage being correlated with better prior performance.

It might be felt that there is perhaps little surprising about the fact that candidates who do well on one examination also do well on another, and so it is worth considering how a low or zero correlation could have been achieved and how it might have been interpreted. Had either of the assessments had a zero reliability (in effect producing random numbers), or been reliable but assessing arbitrary material of no relevance to medicine, then performance of the two assessments would have been uncorrelated. That they are in fact substantially linked supports the idea that both are assessing cognate areas of relevance to medicine. Of course that alone cannot demonstrate validity, for, as has been emphasised earlier, the argument for validity requires information from multiple strands of evidence. The correlation is however compatible with validity, and the argument for validity would be compromised if such a correlation not present.

### Candidate demographics

The candidates taking both MRCGP and MRCP(UK) are clearly not a random or typical subset of the more usual candidates for MRCGP and MRCP(UK), who will normally take just one assessment but not the other. As Table 2 shows, the candidates taking both assessments are different from the more typical candidates taking a single assessment. In general the present sample of candidates perform better at MRCGP than typical candidates, suggesting either that studying for MRCP(UK) has benefited them, or that they were anyway higher-flying or more ambitious candidates. Having said that, they have not performed as well as candidates taking all three parts of MRCP(UK), and the move to MRCGP may have reflected a realisation that they were not likely to succeed as well at hospital medicine, or that their interests were more outside of hospital medicine. But there could also be many other reasons for the undoubted differences including lifestyle changes (including personal relationships, child-rearing and health).

### Differential performance by ethnicity

Having an external measure which is correlated with performance at MRCGP provides a tool for analysing issues which might otherwise be hard or perhaps impossible to assess. The issue of the underperformance of ethnic minority candidates and the relationship between the old and the new CSA examination are good examples of that. BME candidates perform less well at both MRCGP and MRCP(UK). Table 3 also shows that there is an ethnicity effect in both MRCGP and MRCP(UK) at each stage of each examination, BME candidates performing less well even after taking performance at previous stages into account. The explanation for such effects is not clear, but the fact that the effects occur across two independent examinations, in both MCQ and clinical examinations, and after taking previous performance into account, suggests that the effects are unlikely to be due to particular features of any one assessment, component of an assessment or style of assessment.

A similar effect has been reported in several cohort studies, ethnic minorities underperforming at successive stages, even after taking previous performance into account by structural equation modelling. Detailed studies of both MRCGP and MRCP(UK) suggest that differences in performance of BME candidates are unlikely to be due to bias on the part of clinical examiners, in part because differences also exist for MCQ assessments, and because marks awarded seem to show only very small relationships to ethnicity of examiner interacting with ethnicity of candidates [10,28,29].

The differences between the old and the new CSA assessment are of interest, the new CSA having been introduced with the intention of producing a more valid, more reliable assessment of clinical skills, although there have been concerns that this might not be the case particularly for some sub-groups [30]. The analyses of Table 4 show that, for the knowledge examinations, the correlation of MRCGP AKT and MRCP(UK) Parts 1 and 2 are almost entirely identical for white and BME candidates. The relationship between CSA and PACES is not the same, however, in the various groups. As described earlier, PACES correlates more highly with the new CSA than with the old CSA, suggesting that the new examination is a more valid assessment. BME candidates also show a higher correlation between CSA and PACES than do white candidates, which suggests that there is *less* extraneous variance within the BME candidates, making it a more valid assessment. Correlations can be higher because of a greater range of marks, but that is not the explanation for the present data since the regression analysis (see Figure 1), suggests that the regression slopes of CSA on PACES are steeper for BME candidates than for white candidates.

The regression also assessed whether there was an interaction between ethnicity and the old and new CSA assessments, and there was no evidence of an effect either on the intercept or the slope. If the new CSA were unfairly biased against BME candidates then an interaction would be expected, and the present data therefore do not support any suggestion of bias.

## Conclusions

An important general conclusion of this cross-comparison study is that there are high correlations between attainment at MRCGP and MRCP(UK), providing support for the validity of each assessment, with correlations being particularly strong between similar sub-components (MCQ to MCQ, OSCE to OSCE).

In terms of the more specific issues of differential attainment by ethnicity, differential performance on the MRCGP assessments in terms of candidate ethnicity is predicted and confirmed by the same candidates’ performance on the MRCP(UK) assessments. Old and new CSA formats show broadly similar patterns of correlation with MRCP(UK) results, with those from the new format being somewhat higher.

The current analyses have shown that additional value can be added to analyses of postgraduate examination performance by combining data from several colleges or examination boards, to contrast the performance of those taking both assessments.

Although the numbers doing so may be small compared with the numbers taking only a single assessment, they are still large enough in the case of two large examinations such as MRCGP and MRCP(UK) to achieve substantial sample sizes: this allows detailed analysis which can contribute to an understanding of the behaviour of both assessments, and make an additional contribution to arguments for the validity of each. In doing so, they respond perhaps to requests for ‘interdisciplinary’ studies towards the goal of fairness in postgraduate medical assessment [31].

## Abbreviations

AKT: Applied Knowledge Test (in MRCGP)
BME: Black and minority ethnic
CSA: Clinical Skills Assessment (in MRCGP)
GMC: General Medical Council
GP: General practitioner
IRT: Item response theory
MCQ: Multiple choice questions
MRCGP: Membership of the Royal College of General Practitioners
MRCP(UK): Membership of the Royal Colleges of Physicians of the United Kingdom
NHS: National Health Service
OSCE: Objective structured clinical examination
PACES: Practical Assessment of Clinical Examination Skills (in MRCP(UK))
PLAB: Professional Linguistics and Assessment Board (of the GMC)
W: White
